# 

**Published:** 1856-07-01

**Authors:** 


### CONTENTS.

### PART I.—ORIGINAL COMMUNICATIONS.

PAGE
1. The Peace . . . . . . . . . . . . . . . . . . . . . . 312
2. Psychology of Leibnitz . . . . . . . . . . . . . . . . . 325
3. Notes of a Visit to the Public Lunatic Asylums of Scotland . . . 352
4. On the Physiological and Psychological Phenomena of Dreams and Apparitions . . . . . . . . . . . . . . . . . . . . . 373
5. On the Connexion between Morbid Physical and Religious Phenomena 385
6. Physiological Psychology . . . . . . . . . . . . . . . . . 395
7. William Palmer . . . . . . . . . . . . . . . . . . . . 418

### PART II.—REVIEWS.

1. On Suicide and Suicidal Insanity . . . . . . . . . . . . . . 445

### PART III.—FOREIGN PSYCHOLOGICAL LITERATURE.

1. Relations of Anatomy to Psycheatry . . . . . . . . . . . . 465
2. Dr. Dämerow's Résumé of the Question concerning Monomania, and Remarks upon Doubtful Alienation of Mind . . . . . . . . 468
3. Fanaticism or Insanity? . . . . . . . . . . . . . . . . . 473
4. Comments upon a Case of Murder . . . . . . . . . . . . . 477
5. The Causes of Insanity . . . . . . . . . . . . . . . . . . 479
6. On Goître and Cretinism . . . . . . . . . . . . . . . . . 480
7. Alternating Mania and Melancholy cured by Quinine . . . . . . 482
8. Mania and Delirium—Treatment in several Cases . . . . . . . 483
9. Medico-Legal Cases . . . . . . . . . . . . . . . . . . . 484

Contributions to the Chemistry and Histology of the Urine in the Insane 488
Lunatic Asylums in Ireland . . . . . . . . . . . . . . . . . 497
The "Private Lunacy Committee" and the Commissioners in Lunacy . 500

